# Wound Area Measurement with Digital Planimetry: Improved Accuracy and Precision with Calibration Based on 2 Rulers

**DOI:** 10.1371/journal.pone.0134622

**Published:** 2015-08-07

**Authors:** Piotr Foltynski, Piotr Ladyzynski, Anna Ciechanowska, Karolina Migalska-Musial, Grzegorz Judzewicz, Stanislawa Sabalinska

**Affiliations:** Nalecz Institute of Biocybernetics and Biomedical Engineering, Polish Academy of Sciences, Warsaw, Poland; University of Alabama at Birmingham, UNITED STATES

## Abstract

**Introduction:**

In the treatment of chronic wounds the wound surface area change over time is useful parameter in assessment of the applied therapy plan. The more precise the method of wound area measurement the earlier may be identified and changed inappropriate treatment plan. Digital planimetry may be used in wound area measurement and therapy assessment when it is properly used, but the common problem is the camera lens orientation during the taking of a picture. The camera lens axis should be perpendicular to the wound plane, and if it is not, the measured area differ from the true area.

**Results:**

Current study shows that the use of 2 rulers placed in parallel below and above the wound for the calibration increases on average 3.8 times the precision of area measurement in comparison to the measurement with one ruler used for calibration. The proposed procedure of calibration increases also 4 times accuracy of area measurement. It was also showed that wound area range and camera type do not influence the precision of area measurement with digital planimetry based on two ruler calibration, however the measurements based on smartphone camera were significantly less accurate than these based on D-SLR or compact cameras. Area measurement on flat surface was more precise with the digital planimetry with 2 rulers than performed with the Visitrak device, the Silhouette Mobile device or the AreaMe software-based method.

**Conclusion:**

The calibration in digital planimetry with using 2 rulers remarkably increases precision and accuracy of measurement and therefore should be recommended instead of calibration based on single ruler.

## Introduction

Management of chronic wounds like pressure ulcers or diabetic foot ulcers is time and money consuming therefore the progress of healing should be assessed. It was showed that change of wound surface area over time is a good predictor of wound healing and in the treatment of patients with diabetic foot ulceration there is a recommendation to reevaluate the clinical procedures if the wound does not reduce its area by more than 40% in 4 weeks [1]. Wound area measurement were also used in the prediction of healing of deep pressure ulcers [2], venous leg ulcers [3], and postoperative diabetic foot wounds following partial amputation [4].

Wound surface area is sometimes estimated from the length and width of wound measured with a ruler [5] as an area of rectangle or, in better approximation, as an area of an ellipse. A more accurate method is based on tracing the wound contour on transparent film and then by measuring the area of the tracing, which is made by counting the number of squares within the tracing after placing the film onto a printed grid [6]. In more advanced methods the area of such tracing is measured by planimetric tablet like the Visitrak device[7, 8], or a smartphone software[9]. A more specialized device for wound area measurement uses two laser beams for linear dimensions calibration and skin curvature compensation[10]. The most advanced 3D imaging systems[8, 11, 12] provide more detail analysis of wound geometry, but theirs use in clinical practice is not common due to high costs and time consuming measurements.

The method of wound area measurement using a planimetric software (or a graphical software with appropriate functions) and digital photographs is popular as being easy to perform and inexpensive. The wound is photographed with a ruler or a marker of known dimensions placed at the skin near the wound edge and the image is transferred to a computer and open in planimetric software. The ruler or marker is used for calibration of linear dimensions at the image. Once the wound border is manually traced with a computer mouse the area of wound is calculated and displayed. The free software for wound area measurement can be downloaded from the Internet site of the National Institutes of Health [13].

The wound area measurement with planimetric software has the best accuracy when during the taking of photograph the angle between lens optical axis and wound plane is right. In most cases it is not, because when camera is held in hand it is difficult to set it at right angle to the plane in which the wound is lying. Unfortunately, the more this angle differ from 90° the larger may be the measurement error [14]. Rennert et al. [15] showed that the wound area of 20.1 cm² was underestimated by 7 cm² (34.8%) in the case when camera was not properly positioned e.g. when camera lens axis was not perpendicular to the wound plane. The influence of improper camera positioning does not appear when the imaging device is a flatbed scanner. In this case the skin with wound lies over a scanner glass window and thus the plane with wound is parallel to the scanning plane which assures the best wound reproduction without decreasing its size on the image [16–18].

When a camera is not positioned at right angle to the wound plane the reproduction of wound area at the picture is not the same as when this angle is 90°. The reproduced area is proportional to the cosine of this angle. It would be easy to compensate the influence of this angle if we knew this angle, but we don’t. Moreover, there is another issue with the position of the calibrating ruler which should be placed near the wound edge and visible at the picture. Let’s suppose that camera is oriented not at right angle, but it is positioned at the angle of 85°, and this angle is between lens axis and the lower vertical axis of the photographic plane. In such the case the angle above the image center is 95°, and the angles at right and left sides are 90°. Now, if we have an object in the center of the image and the ruler is below this object, the area of the object measured with digital planimetry will be underestimated. By contrary, in the same camera position, but with the ruler placed above the object, the area will be overestimated. The angle, if it is not 90°, always lowers the area at the photograph in comparison to the area taken from 90° position, but the ruler may be reproduced as larger or smaller depending of its position at the picture. When the ruler is reproduced as smaller, the resulting value of wound area is likely to be overestimated in relation to the true area. As the ruler size reproduction is not the same as the wound size reproduction, the result of measurement is inaccurate.

In order to proper calibrate the linear dimensions at the image we should have two crossed at 90° rulers placed exactly on the wound, then the reproduction of wound area would be as the averaged reproduction of linear dimensions of two rulers. A good substitution of these two rulers placed on the wound would be four rulers placed around the wound as sides of a rectangle. The averaged linear dimensions from all rulers could be used in accurate wound area measurement. We tried to use this procedure, but the time for single measurement had remarkably extended. Moreover we noticed that the linear dimensions taken from two rulers placed at the left and right sides of the wound did not improved significantly the final result, and as the calibration of these two additional rulers extended time of measurement, we decided to study the impact of the use only two rulers placed below and above the wound for accuracy and precision of wound area measurement in comparison to the use of single ruler in the measurement.

The aim of the present study is to present a comparison of an advanced and the standard procedure of calibration in wound area measurement with digital planimetry. The proposed advanced procedure of calibration is based on two rulers and the results of area measurements with this procedure are compared to the results from other methods of wound area measurement.

## Materials and Methods

The comparison of measurement techniques may be performed when real wounds are measured [16, 17], but in many cases the wound borders are not well defined and it is better to use wound artifacts [5, 18] which dimensions are stable over time and wound borders are very well defined. If it is possible a reference method of measurement should be used and the results from experimental methods compared to the measurement values from the reference method. In the present study wound shapes printed on a paper were the measurement objects and a reference method of area measurement was used.

In the first part of this study accuracy and precision of wound area measurement based on planimetry software and digital photographs were compared for 2 cases: when single ruler or two rulers were used for calibration of linear dimensions. In the second part the results from digital planimetry techniques were compared to the results from two commercial devices: the Visitrak (Smith & Nephew, United Kingdom) and Silhouette Mobile (Aranz Medical Ltd., New Zealand) and to the results from the method based on the previously developed AreaMe software (Nalecz Institute of Biocybernetics and Biomedical Engineering, Polish Academy of Sciences, Poland) [9].

### Wound Artifacts

Forty wound artifacts (see S1, S2 and S3 Figs) were prepared which were the wound shapes printed on separate sheets of white paper. The wound shapes were created in CorelDraw (Corel Corp., Ottawa, Canada) based on wound images from our previous study [19] or found in an Internet search. Each wound shape was a grey wound outline filled with grey color. The reference area of each wound shape was determined from its images obtained with optical scanner in resolution of 600 x 600 dpi, and saving as black-and-white bitmap. The wound shape on such an image was black and the background was white, therefore, counting the black pixels in the Corel PaintShop Pro using the histogram function, and multiplying its number by the area of a single pixel we received the area of each wound shape. The area of a single pixel was determined from the scanner resolution, and for 600 dpi x 600 dpi it was 1.792·10^−5^ cm². The range of wound shape areas was from 0.14 to 31.72 cm², and the wound shapes were divided into 4 subgroups: very small (below 1 cm²), small (1–2 cm²), medium (2–8 cm²), and large (> 8 cm²).

### Taking a photograph

**Fig 1:**
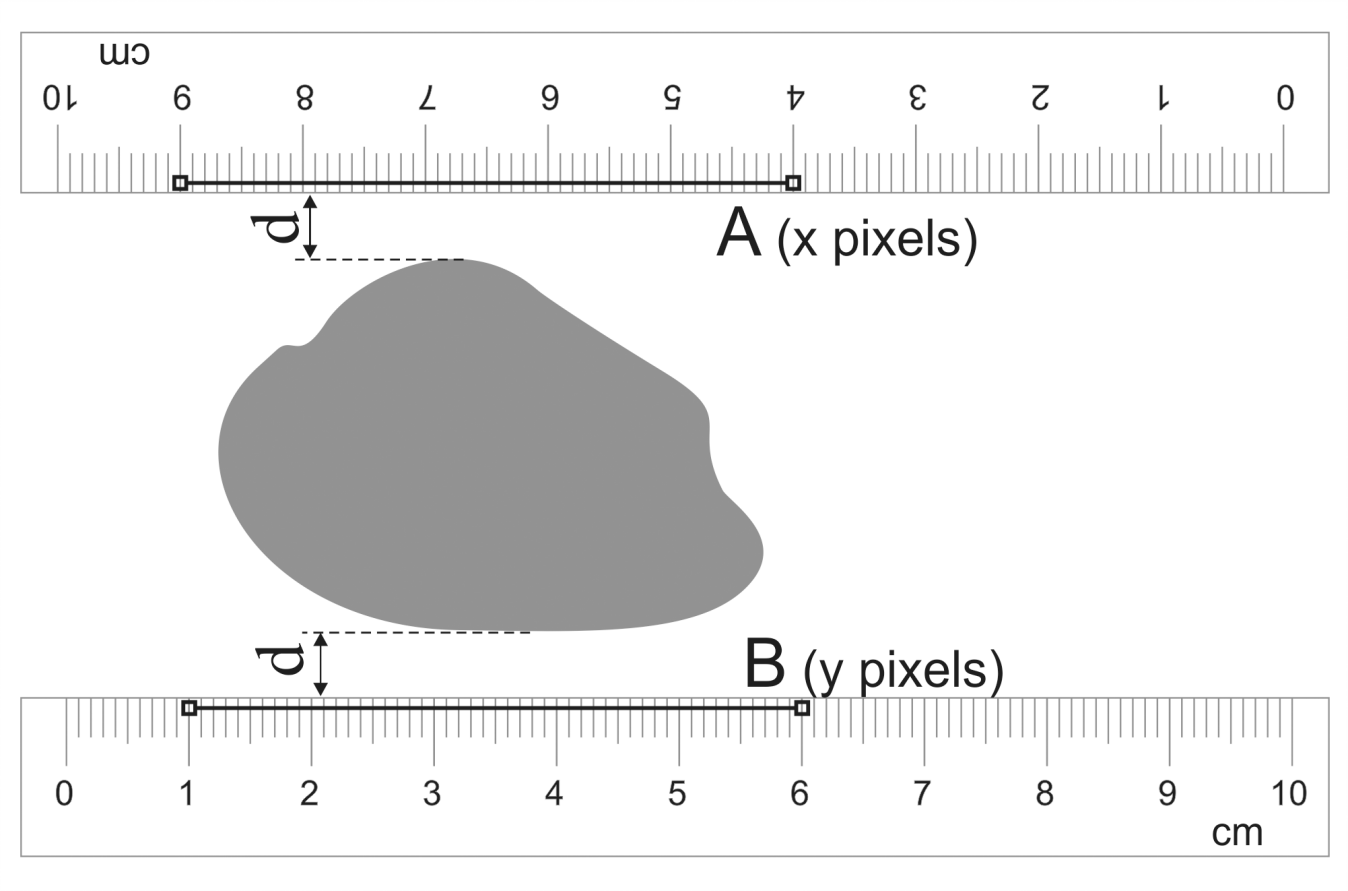
Two rulers placed above and below the wound at approximately equal distances (*d*) from the wound edges. There are 2 line segments (denoted as A and B) of the same length in cm drawn at the upper and the lower ruler which are used in wound area measurement for linear distance calibration.

Printed sheet of paper with the wound shape was placed in a support similar to standard photo frame and a paper ruler sticker was attached below or above the wound shape. In the enhanced procedure (two ruler technique) there were 2 ruler stickers placed at both sides (usually at top and bottom) of the wound shape. The rulers were placed parallel to each other and at short distance (5–10 mm) from the wound shape edge (Fig 1). In each ruler position a separate photograph of wound shape and ruler or rulers was taken. Each wound shape was photographed 3 times with 3 cameras: a compact digital camera (point-and-shot camera), a digital single-lens reflex camera (D-SLR), and a smartphone camera. We used the 14 megapixel D-SLR Nikon D3100, the 5 megapixel compact Canon PowerShot A610, and the 13 megapixel Samsung Galaxy S4 cameras. The position of the camera was no closer than 30 cm apart from the wound shape and the wound shape was visible in the middle of the picture. The picture was taken after focusing onto the wound shape. An optical zoom-in function was used in the point-and-shot and the D-SLR cameras in order to narrow the viewing angle of lens, to get lower spatial distortion, and to better fill the image with main objects e.g. wound shape and rulers. The smartphone camera had only digital zoom-in function, but it was not used.

Although there is an influence of the camera angle and the ruler position on the result of area measurement this angle was not controlled or measured. An operator kept the camera in his hands and tried to find such a camera view to take a picture perpendicularly to the wound shape plane. There was no kind of support in positioning the camera to achieve an angle close to right angle. By that approach the real circumstances in which the photographs are usually taken were maintained. The angle at which a photograph was taken was random factor in the measurement.

### Area measurement with digital planimetry software

The area measurement was performed using ImageJ software available from the Internet site [13] of the National Institutes of Health. The calibration of linear dimensions in the image was made using the tool “Straight line” after a line segment was drawn along the ruler. The length of the line segment was corresponding to the wound shape size, but its length was 2 cm in the case of small wound shapes and it was larger (up to 7 cm) for larger wound shapes. Then using the “Set Scale” function, the “Known distance” was entered as the length of the drawn line segment and the software recalculated the number of pixels in the drawn line segment into the number of pixels per 1 cm.

In two ruler technique (see Fig 1) it was necessary to draw a line segment at first ruler, choose “Set Scale” from the “Analyze” menu and note the number of pixels from the field “Distance in pixels”. Then draw another line segment of the same number of centimeters at the second ruler and note the number of pixels in this line segment, calculate the arithmetic mean from these two numbers of pixels and then enter the result using keyboard in the field “Distance in pixels”; the “Known distance” in this case was the length of the drawn line segments.

Next, the wound outline was created using the “Polygon selections” tool and manual tracing of the wound shape edges with a computer mouse. The area was calculated in cm² after choosing “Measure” from the “Analyze” menu.

The area measurements of the wound shapes were performed in random order, and the operator was not aware of the wound shape classification.

### Other methods of wound area measurement

All wound shapes were also measured with Visitrak device, Silhouette Mobile and the AreaMe software developed for smartphones with Windows Mobile operating system [9]. The Visitrak is a digital planimetric tablet measuring wound area after retracing the wound outline with an enclosed stylus. The wound outline need to be traced earlier on a double-layer film placed on a wound. The Silhouette Mobile device is a combo device composed of a PDA computer and a head that emits two laser beams and takes photographs. Software on the PDA uses displayed lines at skin to calibrate linear dimensions and calculates the wound area after manual tracing of wound outline. The AreaMe software calculates automatically the wound area after taking a photograph of the wound outline traced earlier manually on transparent film and placed over a 1 x 1 cm grid.

### Statistical analysis

The accuracy of the measurement techniques was assessed by analyzing relative errors (REs). The RE was calculated as a ratio of absolute difference between the measured and actual values to the actual value. The actual value was measured with a reference method. For the wound shapes the area measured with by the pixel counting method in the images from optical scanner (described above) was the reference value. Mann-Whitney test and Kruskal-Wallis one-way analysis of variance by ranks were used for comparison of the REs.

The precision of measurement techniques, also called repeatability, was assessed by comparing standard deviations (SDs) of relative differences (RDs). The RD was calculated as ratio of difference between the measured and actual values to the actual value. The lower SD of RDs, the higher the precision (repeatability). Analysis of variance (ANOVA) and Kruskal-Wallis one-way analysis of variance by ranks were used to compare means or medians, respectively. The SDs of RDs for each technique were compared and tested using tests for homogeneity of variances: F-test, Bartlett’s, Cochran’s, Hartley’s, and Levene’s tests. Normality of data were tested using Kolmogorov-Smirnov, Lilliefors, and Shapiro-Wilk tests for normality. A p-value lower than 0.05 was considered statistically significant.

## Results

**Fig 2:**
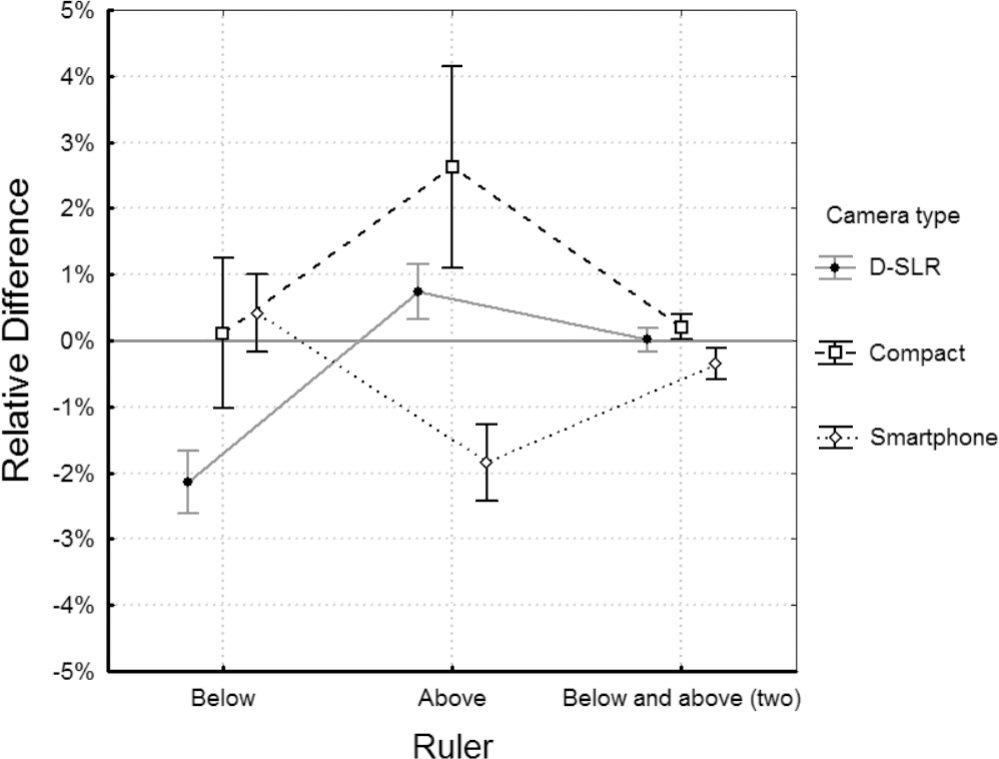
Relative differences between the measured area and reference area expressed in percentage for the digital planimetry based on photographs taken by 3 types of digital cameras in 3 positions of ruler: below the wound shape, above the wound shape, and with 2 rulers placed below and above. Vertical bars denote 95% confidence intervals.

Analysis of variance in digital planimetry method with one or two rulers revealed that the position of ruler is a significant parameter influencing the RD (p = 0.008). The tests of homogeneity of variances showed significant (p < 0.005) differences for ruler position for each camera type. The detailed comparisons showed that the SDs for two ruler technique are significantly smaller than the SDs for one ruler placed below or above the wound shape (Fig 2). On overall, the SD for two ruler technique was equal to 0.64% and it was 3.8-fold lower than the overall SD for the one ruler technique (SD = 2.46%). Table 1 shows ratios of mean SD of the one ruler techniques and SD of two ruler technique for all camera types. In the case of the D-SLR and the smartphone cameras the improvement in precision is 2.4-fold, and 7.1-fold in the case of the compact camera.

**Table 1:** Standard deviations and ranges of relative differences between measured area and true area for three camera types and ratios of mean SDs for one ruler techniques and two ruler technique. **N denotes number of measurements**.

Camera type	SD (range) of relative differences			Ratio of mean SD for one ruler techniques and SD for two ruler technique
	Ruler below	Ruler above	Two ruler technique	
**D-SLR**	1.48% (-5.1–1.8)%, N = 40	1.30% (-3.0–3.7)%, N = 40	0.58% (-1.6–1.2)%, N = 40	2.4
**Compact**	3.56% (-6.9–9.0)%, N = 40	4.75% (-10.0–11.2)%, N = 40	0.59% (-1.4–1.3)%, N = 40	7.1
**Smartphone**	1.85% (-2.4–6.6)%, N = 40	1.80% (-7.2–1.8)%, N = 40	0.75% (-1.7–1.2)%, N = 40	2.4
**Overall**	2.30%, N = 120	2.62%, N = 120	0.64%, N = 120	3.8

**Fig 3:**
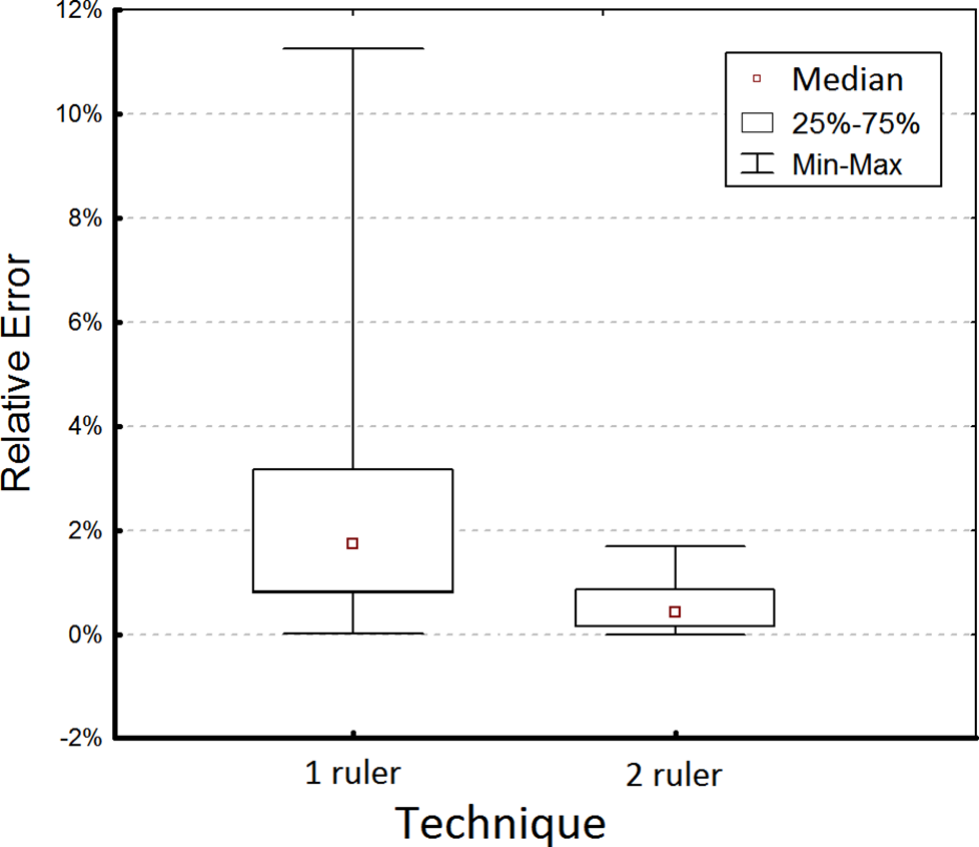
Box plot of relative errors of area measurement expressed in percentage for the digital planimetry based on one ruler or two ruler calibration techniques.

In order to compare the overall accuracy of the one ruler technique (with ruler placed below or above the wound shape) to the two ruler technique the REs were compared using the Mann-Whitney test and the results were significant with p < 0.0001. The data from all 3 cameras were included to this analysis. Fig 3 shows the box-plot of REs for these two techniques. The medians of REs were 1.73% and 0.43% for the one ruler and two ruler techniques, respectively. The median of REs for the one ruler technique was 4.02 times greater than that for the two ruler technique, therefore on average, the accuracy of the two ruler technique is 4 times greater.

In the next analysis the wound area range was a factor tested for influencing the precision of the digital planimetry with 2 rulers. Data from all 3 cameras were included. Differences between SDs of RDs were tested using the analysis of homogeneity of variances and the test results were nonsignificant with p > 0.22. The precision of digital planimetry with two rulers do not change with the change of measured area and the SDs of RDs were 0.71%, 0.58%, 0.71%, 0.55% for very small, small, medium and large wound area range, respectively.

Similarly, in the next analysis the camera type was a factor tested for influencing the precision of the digital planimetry with 2 rulers. As the distributions of RDs were normal and the sample sizes were equal, homogeneity of variances were tested using Hartley’s *F*_*max*_ test and the result was p = 0.198. This means that the precision of digital planimetry with 2 rulers is not camera-type dependent.

**Fig 4:**
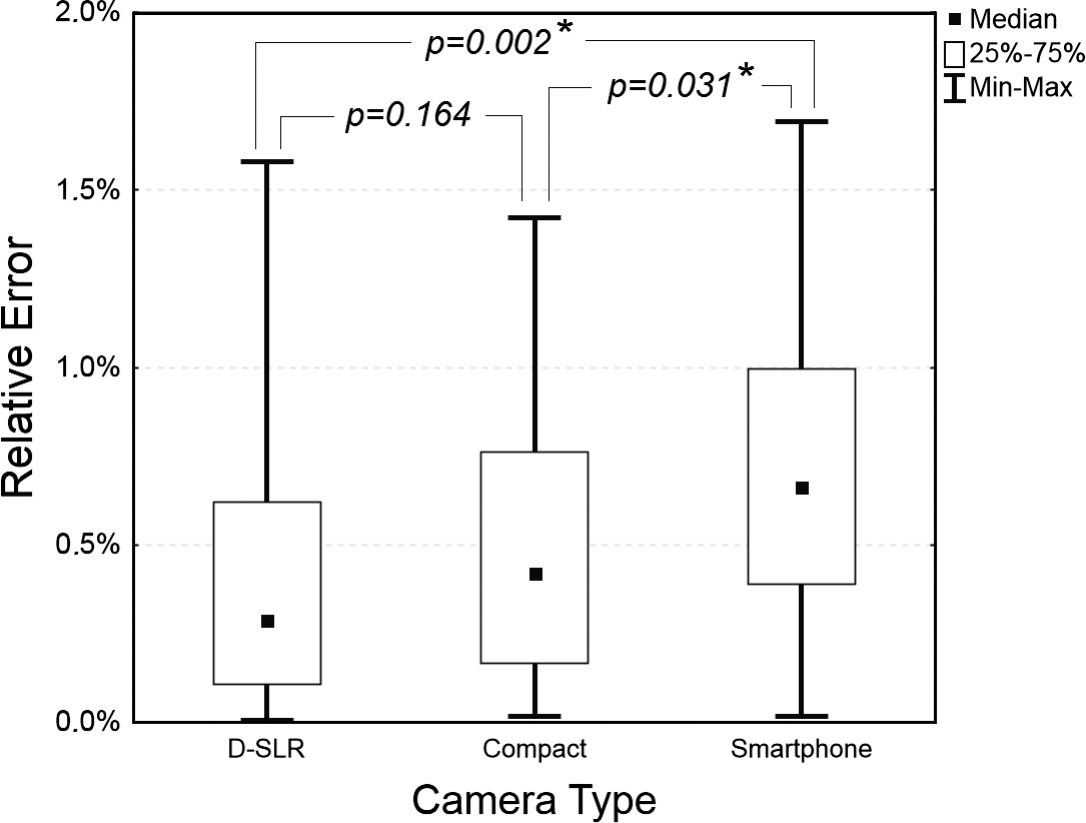
Box-plot of relative errors of area measurement expressed in percentage for the digital planimetry method based on 2 rulers for 3 camera types: D-SLR, Compact, and Smartphone. An asterix denotes statistically significant difference between the medians.

The influence of the camera type on the accuracy of digital planimetry with 2 rulers was tested by comparing medians of REs. The result of the Kruskal-Wallis test was significant with p = 0.005. Fig 4 shows box-plot of REs with p-values of detailed analysis in which each camera was compared with each other. The insignificant difference was between the D-SLR and the compact cameras (p = 0.164), and significant differences were between the smartphone camera and each of the two other cameras with p = 0.002 and p = 0.031 for the D-SLR and the compact camera, respectively.

**Fig 5:**
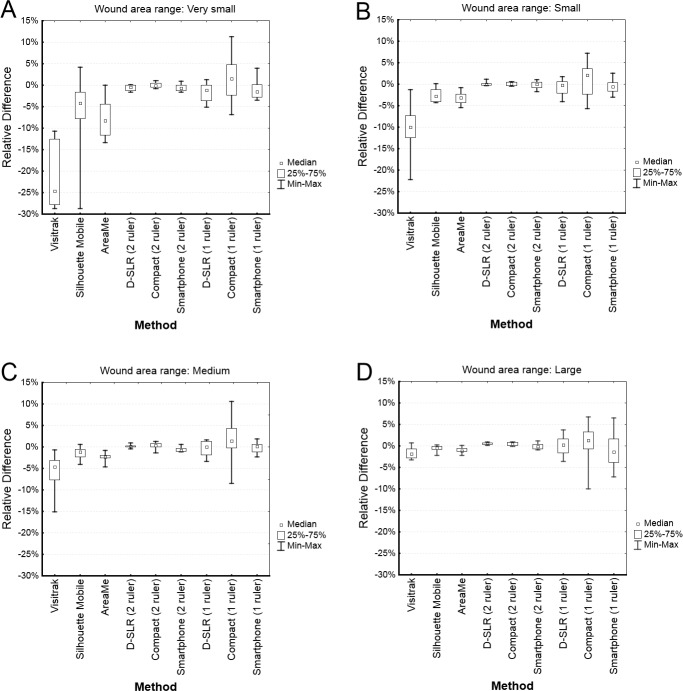
Box plots of relative differences between the measured area and reference area expressed in percentage for the Visitrak device, the Silhouette Mobile device, the AreaMe software and for the digital planimetry methods based on two ruler calibration in 4 ranges of wound area: (A) very small (< 1 cm²), (B) small (1–2 cm²), (C) medium (2–8 cm²), and (D) large (> 8 cm²).

The precision of digital planimetry with two rulers was subsequently compared with the Visitrak device, the Silhouette Mobile device, and the AreaMe software. The tests were performed in 4 wound area ranges: very small, small, medium, and large. Fig 5 shows box plots of RDs for the included methods in these area ranges. The Kruskal-Wallis test revealed significant differences (with p < 0.0002) between medians of RDs in the set of area measurement methods for each wound area range and also for data without dividing into ranges.

**Fig 6:**
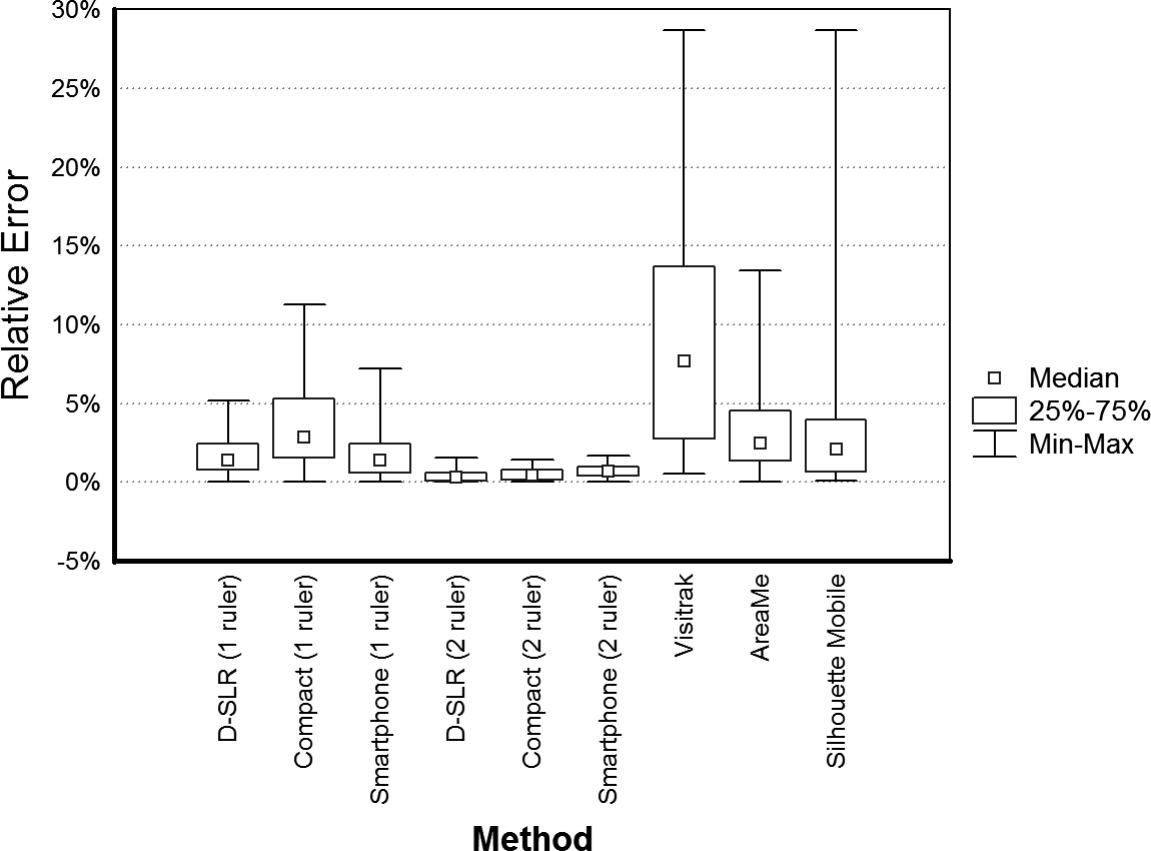
Box plot of relative errors expressed in percentage for the digital planimetry methods (based on one and two ruler calibration) and for the Visitrak device, the Silhouette Mobile device and the AreaMe software. The differences between medians are significant with p < 0.0001.

The accuracy of digital planimetry with two rulers based on all camera types was compared with accuracies of the Visitrak device, the Silhouette Mobile device, and the AreaMe software. There were significant differences (with p < 0.0001) between medians of REs in the set of area measurement methods for the very small, small and medium wound area range and for all data without dividing into ranges (Fig 6), but there was no difference in accuracy in the large wound area range (p = 0.075).

## Discussion

The results of the statistical tests revealed some important advantages of the digital planimetry with two rulers over planimetry with single ruler. Precision of area measurement with digital planimetry based on two rulers was 2.4 to 7.1 fold higher than based on single ruler. Analysis of graph in Fig 2 reveals also that measurements based on one ruler calibration are prone for bias as the confidence intervals do not contains zero. Such a systematic error could be caused by the camera operator who was skewing the camera view in the same direction during taking of photographs. The same error could appear in the results from the two ruler technique, but it is not present for the D-SLR camera and it has small magnitude for the other types of camera as their confidence intervals are close to zero. The two ruler technique is more resistant to the bias, which is very important in the measurements, because it increases the accuracy of measurement.

The accuracy of measurement based on the two ruler technique is independent of camera type and this is probably due to: (a) the use of optical zoom function in the D-SLR and compact cameras and (b) taking the measurements with a distance not smaller than 30 cm with the smartphone camera which do not produce as large image distortions (which influence the accuracy) as when the picture is taken at much closer distance. Some of the wound shapes were small and the operator could have an idea to take a picture with closer distance to better fill the picture, but this could decrease the accuracy.

Precision of area measurement with digital planimetry based on two rulers is significantly higher than precision of measurement with one ruler, because SDs of RDs were significantly smaller in the case of the two ruler technique in comparison to the standard technique with one ruler. This result was independent of camera type used for taking the photographs. The overall precision of the two ruler technique was 3.8 times larger (Table 1) than the one ruler technique, and in practice we didn’t get deviation from the true value of area larger than 1.7% for the two ruler technique in contrast to the one ruler technique where the maximal deviation was 11.2% (Table 1). This was observed in 120 measurements with the two ruler calibration and in 240 with the one ruler calibration.

The digital planimetry with two rulers was also tested using the area range as a parameter. This technique had best accuracy in the area range of small and medium, but it underestimated the measured area for very small areas, and overestimated for large areas, however the mean absolute value of overestimation or underestimation was very small of about 0.4%. When the influence of area range on the precision of the digital planimetry with two rulers was tested the result was insignificant which means that precision of area measurement using this technique is independent of wound area.

The influence of camera type on the precision of area measurement with digital planimetry with two ruler calibration was also tested and the results were nonsignificant. This is very important for the users of smartphones, because even with lower quality of smartphone cameras the precision of measurement is not significantly lower.

In the next analysis the camera type was the tested parameter and it was checked whether it influences the accuracy of digital planimetry with two rulers. The result of the test was significant, and the measurement based on the D-SLR camera was the most accurate and based on the Smartphone camera the least accurate. This result is the most probably due to the low image distortions of the D-SLR camera and larger image distortions in the smartphone camera. As the D-SLR camera assures the best accuracy, this type of camera should be chosen for clinical research.

The digital planimetry with two rulers, the Visitrak device, the Silhouette Mobile device, and the AreaMe software were compared in 4 area ranges revealed significant differences in accuracy of these methods except for the wound areas larger than 8 cm² in which the medians of relative errors were not significantly different. The smaller the measured area was the larger was inaccuracy for the Visitrak, the Silhouette Mobile and the AreaMe software. The accuracy of these 3 methods was much lower than accuracy of digital planimetry with two rulers. In the case of very small wounds with areas below 1 cm² the number of digits after decimal point in the result may be responsible for low overall accuracy in the case of the Visitrak and Silhouette Mobile devices as they display only one digit in the result. When, for instance, the true area is 0.149 cm², the devices may measure it and round the result to either 0.1 or 0.2 cm² and then the calculated RDs are -32.9% and 34.2%, respectively. The AreaMe software displays two digits after decimal point therefore the RDs caused by rounding are lower for this method. The Visitrak and the Silhouette Mobile devices should not be chosen for the measurement of very small wounds. The low accuracy of the Visitrak device in the measurement of small wounds was earlier shown in two studies [18, 20].

The results of the current study indicate that the use of 2 rulers for calibration in digital planimetry efficiently improves accuracy and precision of area measurement. The two ruler technique remarkably reduces variation of area measurement by averaging the number of pixels per 1 cm from two sides of the measured wound. This averaging may be compared to the calibration with using a virtual ruler lying directly over the center of measured wound. Each of the rulers calibrate one axis (horizontal) and the difference in the number of pixels per 1 cm from these two rulers is a measure of skewnees in the perpendicular axis (vertical). This way the entire region with wound is taken into account for calibration.

A square paper marker placed near the wound and photographed with it was used by Shetty et al. [21] for calibration in wound area measurement. They proposed using the number of pixels per 1 cm² for calibration, which could be determined from the number of pixels in the 16 cm² square. Unfortunately, the use of only one square marker at one side of the wound is almost the same as using one ruler for calibration at one side. Two squares placed at opposite sides of wound would help much more, but they did not suggest such a technique. The technique with one square marker was not compared with standard technique based on one ruler commonly used for calibration and possible superiority of calibration with square marker was not confirmed.

Based on the results of the current study one can conclude that digital planimetry with two rulers will be the best method, of all presented in this study, for small wounds and for wounds placed at skin region with low curvature. The other methods presented here will have better accuracy on curved skin, but they are also not suitable for all cases. There are regions of skin where the wound area measurement is always problematic. For example, a large wound around heel will cause problems, because transparent film used in the Visitrak device and in the AreaMe method will not cover properly such a region and the wound tracing will be made with errors. Measurement at heal with the Silhouette Mobile device will be also problematic as it requires some region of healthy skin in order to proper compensate the skin curvature. Every non 3D method in such a region will measure the area with certain approximation.

The main premise of the current study results is as follows: when digital planimetry based on digital pictures is used for wound area measurement the calibration of linear dimensions based on 2 rulers will assure higher accuracy and precision than based on single ruler.

## Supporting Information

S1 Fig Wound shapes (#1—#20) used in the study. (PDF)

S2 Fig Wound shapes (#21—#39) used in the study. (PDF)

S3 Fig Wound shape (#40) used in the study. (PDF)
