# Supplementary material for: Wound Area Measurement with Digital Planimetry: Improved Accuracy and Precision with Calibration Based on 2 Rulers
Source: PLoS One. 2015 Aug 7;10(8):e0134622. doi: 10.1371/journal.pone.0134622 (PMC4529141; doi:10.1371/journal.pone.0134622)
Supplement: S1 Fig — (PDF) [file pone.0134622.s001.pdf]

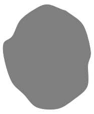
#1 (1.215 cm²)

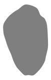
#2 (0.411 cm²)

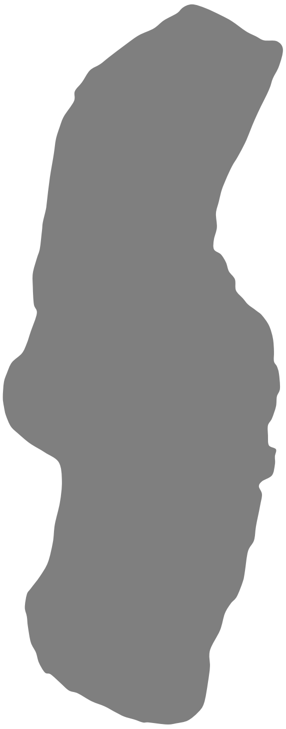
#3 (24.027 cm²)

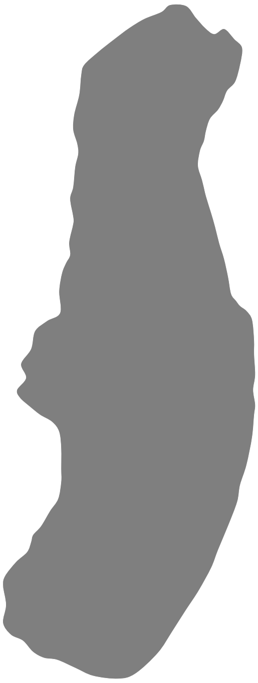
#4 (19.275 cm²)

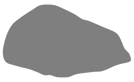
#5 (1.109 cm²)

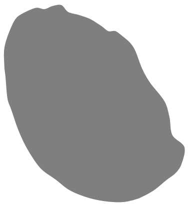
#6 (4.313 cm²)

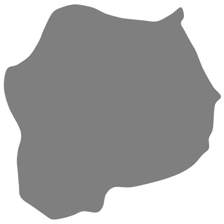
#7 (5.787 cm²)

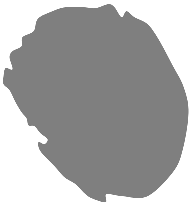
#8 (4.593 cm²)

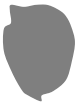
#9 (0.915 cm²)

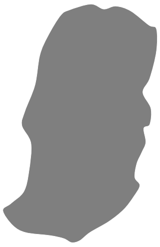
#10 (4.374 cm²)

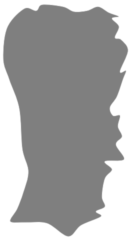
#11 (3.746 cm²)

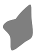
#12 (0.140 cm²)

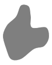
#13 (0.260 cm²)

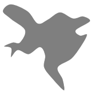
#14 (0.542 cm²)

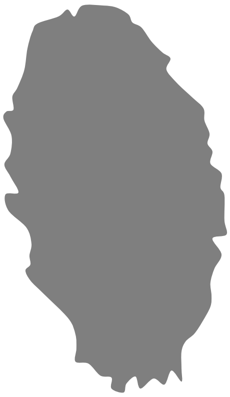
#15 (10.651 cm²)

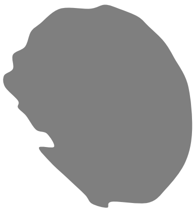
#16 (4.744 cm²)

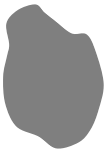
#17 (1.878 cm²)

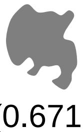
#18 (0.671 cm²)

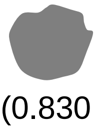
#19 (0.830 cm²)

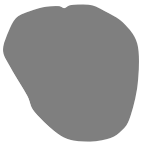
#20 (2.502 cm²)
