# Supplementary material for: Wound Area Measurement with Digital Planimetry: Improved Accuracy and Precision with Calibration Based on 2 Rulers
Source: PLoS One. 2015 Aug 7;10(8):e0134622. doi: 10.1371/journal.pone.0134622 (PMC4529141; doi:10.1371/journal.pone.0134622)
Supplement: S2 Fig — (PDF) [file pone.0134622.s002.pdf]

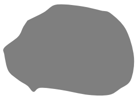
#21 (1.599 cm²)

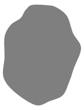
#22 (1.029 cm²)

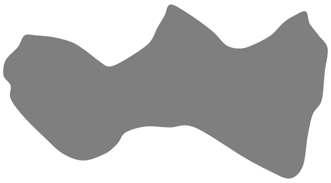
#23 (5.848 cm²)

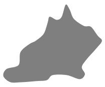
#24 (0.674 cm²)

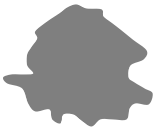
#25 (1.942 cm²)

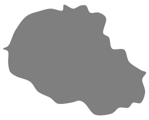
#26 (1.776 cm²)

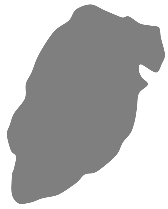
#27 (3.417 cm²)

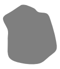
#28 (0.420 cm²)

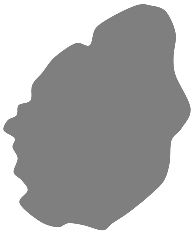
#29 (4.951 cm²)

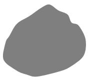
#30 (0.744 cm²)

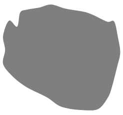
#31 (1.667 cm²)

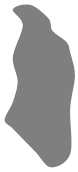
#32 (1.342 cm²)

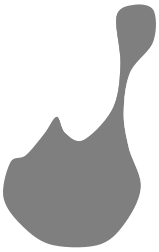
#33 (2.718 cm²)

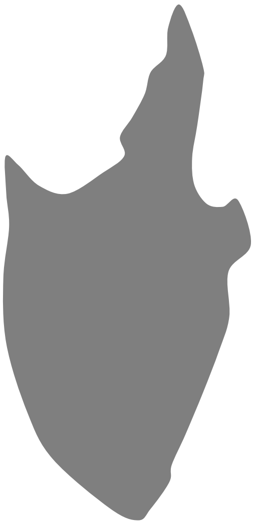
#34 (12.367 cm²)

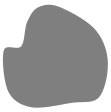
#35 (1.442 cm²)

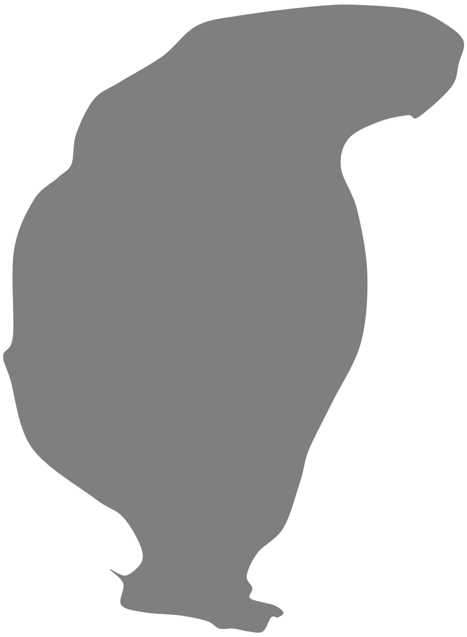
#36 (29.599 cm²)

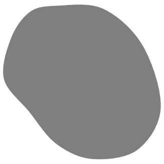
#37 (3.219 cm²)

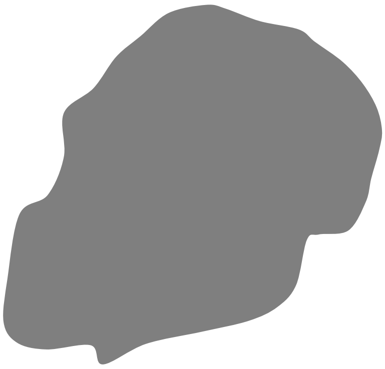
#38 (15.962 cm²)

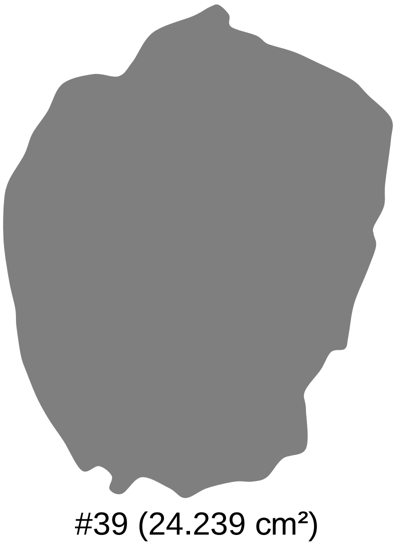
#39 (24.239 cm²)
