# Supplementary figures and images for: Wound Area Measurement with Digital Planimetry: Improved Accuracy and Precision with Calibration Based on 2 Rulers
Source: PLoS One. 2015 Aug 7;10(8):e0134622. doi: 10.1371/journal.pone.0134622 (PMC4529141; doi:10.1371/journal.pone.0134622)

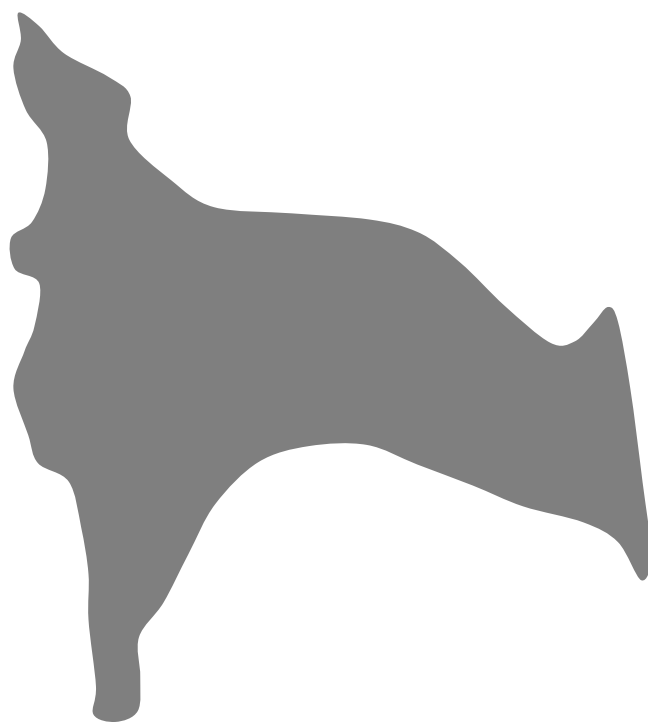

#40 (31.718 cm<sup>2</sup>)

Supplement: S3 Fig — (PDF) [file pone.0134622.s003.pdf]
